# Alterations in acetylcholinesterase activity and oxidative stress parameters induced by pure cylindrospermopsin in brain of orally exposed rats and determination of potential metabolites

**DOI:** 10.1007/s00204-025-04057-5

**Published:** 2025-04-13

**Authors:** Cristina Plata-Calzado, Ana I. Prieto, Ana M. Cameán, Angeles Jos

**Affiliations:** Area of Toxicology, Faculty of Pharmacy, Universidad de Sevilla, Profesor García González 2, 41012 Seville, Spain

**Keywords:** Acetylcholinesterase, Cylindrospermopsin, Metabolites, Neurotoxicity, Oxidative stress

## Abstract

Cylindrospermopsin (CYN) is a secondary metabolite produced by cyanobacteria. In view of its toxic effects, CYN is frequently classified as a cytotoxin, although it has been associated with neurotoxicological effects. Despite this, few studies have explored the impact of CYN on the nervous system and none of them have been conducted in mammals. Therefore, the objective of the present work was to evaluate the effect of different doses of pure CYN (7.5–75.0 µg/kg body weight) on the brain of rats after gavage exposure for 48 h. For this purpose, the activity of acetylcholinesterase (AChE) enzyme and different biomarkers of oxidative stress were assessed. A significant inhibition of AChE activity was observed at all doses tested. Additionally, the results showed a dose-dependent increase in lipid peroxidation (LPO) levels, as well as alterations in superoxide dismutase (SOD) and catalase (CAT) activities. These changes could be produced by CYN metabolites detected in brain samples by ultra-high-performance liquid chromatography coupled to a tandem mass spectrometry system (UHPLC-MS/MS). These findings suggest that metabolites of pure CYN could cause alterations in the brain of rats after oral exposure and highlight the need for further studies focusing on CYN’s neurotoxicity and its potential role in neurodegenerative diseases.

**Supplementary Information:**

The online version contains supplementary material available at 10.1007/s00204-025-04057-5.

## Introduction

Cyanobacteria or “blue-green algae” are prokaryotic organisms whose presence is increasing due to water eutrophication and climate change. Its importance lies mainly in its potential ability to produce harmful secondary metabolites named cyanotoxins (Plaas and Paerl 2021). Cylindrospermopsin (CYN) is one of the most important cyanotoxins to consider due to its worldwide distribution and toxicity. CYN is a tricyclic alkaloid of zwitterionic nature and 415 Da molecular weight. Humans are potentially exposed to CYN by different pathways, such as dermal contact and inhalation during recreational activities, or oral route as a consequence of consuming contaminated water or food (fish, molluscs, vegetables, or algae-based supplements) (Codd et al. 2020; Mutoti et al. 2022).

This cyanotoxin exerts its toxic effects primarily through the suppression of protein synthesis, the production of oxidative stress, and the potential to produce DNA damage following metabolic activation by cytochrome P-450 enzymes (Zegura et al. 2011; Yang et al. 2021). Although the liver is considered the main target organ for CYN toxicity, several research have shown that this cyanotoxin can also affect other organs (Gutiérrez-Praena et al. 2012), including the nervous system. However, studies focusing on the neurotoxicity of pure CYN are scarce and mainly extracts of CYN-producing cyanobacteria have been used (Hinojosa et al. 2019a). In particular, studies on snails (Kiss et al. 2002), toads (Kinnear et al. 2007; White et al. 2007), fish (Guzmán-Guillén et al. 2015a; da Silva et al. 2018) and mice (Saker et al. 2003) have revealed neurotoxic effects after exposure to CYN-producing *Cylindrospermopsin raciborskii* or *Aphanizomenon ovalisporum* cultures.

One of the essential enzymes of the nervous system is acetylcholinesterase (AChE). This enzyme catalyzes the hydrolysis of the neurotransmitter acetylcholine in the synaptic cleft. When AChE activity is suppressed, an accumulation of acetylcholine is produced, resulting in excessive cholinergic stimulation and subsequent neurotoxicity. The alteration of AChE has been reported in rats after exposure to different xenobiotics, such as pesticides (Coban et al. 2015; Chen et al. 2023), drugs (Mowaad et al. 2023) or metals (Carageorgiou et al. 2005), supporting that AChE activity could be a useful biomarker to detect exposure to pollutants.

Likewise, the brain is also especially sensitive to oxidative stress because of its high oxygen consumption, elevated lipid content, and relatively weak antioxidant defences (Bélanger et al. 2011). Moreover, several studies have highlighted the importance of oxidative stress as a mechanism of CYN-mediated toxicity (Gutiérrez-Praena et al. 2011; Puerto et al. 2011; Yang et al. 2021). Enzymatic activities such as superoxide dismutase (SOD) and catalase (CAT) activities, as well as glutathione levels play an essential role in the removal of excess of reactive oxygen species (ROS) and in the preservation of redox balance within cells. When these antioxidant defences are compromised, oxidative damage to biomolecules occurs. In that sense, upon exposure to CYN, an increase in lipid peroxidation (LPO) levels has been observed in hepatocytes of fish *Prochilodus lineatus* (Liebel et al. 2011), liver, kidney and brain of tilapia fish (Puerto et al. 2011; Guzmán-Guillén et al. 2015a) or brain of fish *Hoplias malabaricus* (Da Silva et al. 2018). Specifically, Guzmán-Guillén et al. (2015a) observed that LPO levels in the brain of tilapia fish subchronically treated with CYN were 71% higher than in the control group. Consistent with this, Da Silva et al. (2018) reported a time-dependent increase in LPO levels in the brain of fish *Hoplias malabaricus* exposed to purified CYN and aqueous extract containing CYN administered intraperitoneally (i.p.). Although, as mentioned above, alterations in some parameters of oxidative stress have been observed in combination with changes in AChE activity in the brain of aquatic organisms, the effects in mammals remain uncertain.

Currently, there are no known transporters of CYN through the blood–brain barrier or evidence of passive diffusion (Metcalf et al. 2021); however, two studies have detected the presence of this cyanotoxin in the brain of fish after exposure by i.p. injection (Da Silva et al. 2018) or oral route (Guzmán-Guillén et al. 2015a) by enzyme-linked immunosorbent assay (ELISA). Nevertheless, further research on the possible presence of both CYN and its possible metabolites in the brain of mammals is still needed, as several studies have shown that CYN toxicity may be mediated by metabolites generated by cytochrome P450 (CYP450) (Zegura et al. 2011).

Therefore, this work intends to address this gap by investigating the impact of oral exposure to pure CYN on the AChE activity and oxidative stress parameters in the brain of male Wistar rats orally exposed to pure CYN at three different doses (7.5, 23.7 and 75.0 µg/kg b.w.) for 48 h. Oxidative stress parameters evaluated include malondialdehyde (MDA) levels, a marker of LPO, and the activities of antioxidant enzymes SOD and CAT, as well as oxidized (GSSG) and reduced (GSH) glutathione levels. In addition, to link these toxic effects to pure CYN exposure, ultra-high performance liquid chromatography coupled with a tandem mass spectrometry system (UHPLC–MS/MS) analysis of this toxin and its potential metabolites has been carried out in brain samples.

## Materials and methods

### Chemicals and reagents

Cylindrospermopsin (purity 95%) standard was provided by Enzo Life Sciences (Lausen, Switzerland). C-Viral S.L. (Sevilla, Spain), Sigma–Aldrich (Madrid, Spain), and Gibco (Biomol, Sevilla, Spain) provided all chemicals and reagents used in the assays and analysis performed.

### Animal maintenance

Male Wistar rats (strain RjHan:WI) seven-week-old were purchased from the Centre for Animal Production and Experimentation of the University of Sevilla. Animals were acclimatized for one week prior to the experiment with the following environmental conditions: 23 ± 1 °C temperature, 12-h dark/light cycle, and 55 ± 10% relative humidity and fed and water ad libitum. A standard laboratory diet (Harlan Laboratories, Barcelona, Spain) was used for feeding.

This in vivo experiment has the approval of the Animal Experimentation Ethics Committee of the University of Seville (09/03/2016/028). The animals were cared for according to Directive 2010/63/EU on the protection of animals used in scientific research.

### Experimental design and treatment

Three doses of CYN (7.5, 23.7 and 75.0 µg CYN/kg b.w.) were chosen considering the results previously obtained by other authors (Đorđević et al. 2017; Chernoff et al. 2018) and following the recommendations established in the OECD 474 guideline (OECD 2016). Only male rats were used, in agreement with Chernoff et al. (2018), who reported that were more sensitive to CYN. Animals were acclimatized for a period of 1 week and randomly divided into 4 groups comprising 5 male rats per group: (1) negative control group (C-) administered with water as a vehicle; (2) 7.5 μg CYN/kg exposed group; (3) 23.7 μg CYN/kg exposed group and (4) 75.0 μg CYN/kg exposed group. Doses were administered by gavage at 0, 24 and 45 h and male rats were sacrificed 3 h after the last dose was administered. Subsequently, all brain tissues were removed, weighed, and rinsed with a cold saline solution and stored at −80 °C until analysis. No clinical signs were found during the experiment.

### Sample preparation and quantification proteins

Brain samples were homogenized with ultraturrax in potassium phosphate buffer at 1:10 (w/v) ratio. Then, the homogenates were centrifuged at 10,000 × *g* (10 min at 4 ℃). The supernatants were frozen at −20 ℃ until analysis. The quantification of proteins in the samples was carried out by the Bradford method (1976).

### Activity acetylcholinesterase

The activity of AChE was assessed using the methodology described by Ellman et al. (1961), which was modified for microplate formats. Acetylthiocholine iodide (AcSCh) at a concentration of 9 mM served as the substrate, while 5,5′-dithio-bis-(2-nitrobenzoic) acid (DTNB) at 0.75 mM was the chromogenic agent. The optical density was registered at 415 nm for 3 min every 30 s using a plate reader (TECAN, Infinite® M200, Männedorf, Switzerland).

### Determination of oxidative stress parameters

LPO levels were estimated with the concentration of thiobarbituric reactive substances (TBARS) using the method of Esterbauer and Cheeseman (1990) and adapted for microplate. The results were reported in nmol TBARS per gram of tissue.

The activity of SOD was determined using the xanthine-oxidase-cytochrome C method outlined by McCord and Fridovich (1969). In this process, the interaction between xanthine and xanthine oxidase generated superoxide radicals and the decrease in the reduced cytochrome c was measured using a plate reader. CAT activity was measured following the procedure established by Beers and Sizer (1952). The decrease in hydrogen peroxide concentration was monitored at a wavelength of 240 nm using a spectrophotometer equipped with quartz cuvettes (1.0 mL cuvettes with a light path of 1.0 cm).

GSH and GSSG levels were measured with a kit (Cayman Assay Kit, 703002) following the manufacturer’s instructions.

### CYN extraction and determination by UHPLC-MS/MS

To detect the maximum number of metabolites present in each CYN exposure group, a pool of brain samples from rats that received the same treatment was performed. This also ensured that sufficient samples were available for the subsequent extraction process. The method used for the extraction of CYN is described and validated by Guzmán-Guillén et al. (2015b). Briefly, 10 mL of milli-Q water/acetonitrile was added in a proportion of 30:70 (v/v) with 0.5% TFA (v/v) to a 0.5 g of fresh tissue. Then, the sample was homogenised with ultraturrax (IKA®-WERKE T25 Basic Ultra-Turrax® Homogenizer, Staufen, Germany). Subsequently, the sample was sonicated (15 min) and centrifuged (15 min at 3700 rpm). The resulting supernatant was collected, and the procedure described was repeated. Finally, both supernatants were mixed, obtaining 20 mL of sample.

For the purification procedure, a combined solid phase extraction (SPE) system was used, with a C18 column followed by a porous graphitic carbon (PGC) column following the procedure of Guzmán-Guillén et al. (2015b). The samples were filtered through a 0.22 µm syringe filter before being injected into the UHPLC–MS/MS.

All analyses were performed using a Thermo Scientific liquid chromatography system consisting of a quaternary UHPLC Dionex Ultimate 3000 SD coupled to a quadrupole-orbitrap Q-exactive hybrid mass spectrometer (ThermoFisher Scientific) with heated electrospray ionization (HESI) source. Separation was performed on Acquity HSST3 (2.1 × 100 mm, 1.7 µm) (Waters). 5 µL was used as injection volume and 0.300 mL/min as flow rate. The mobile phase consisted of water with 0.1% formic acid (FA) (Solvent A) and acetonitrile with 0.1% FA (Solvent B). A gradient elution was employed, starting with 0% B (0.0–2.0 min), increasing to 70% B (2.0–12.0 min), followed by 100% B (12.1–13.0 min), and then 0% B up to 15.0 min. The detection was performed in positive mode with full range acquisition over the m/z 100–1000 at a resolution of 70,000 and data-dependent scan TOP5. The following HESI source parameters were used: lens level 50 V spray voltage 3500 V, sheath and auxiliary gas flow (N_2_) 45 and 12.5 (arbitrary units) capillary temperature 320 °C, and probe heater temperature 350 °C. Data acquisition and data analysis were managed through Xcalibur software.

In parallel, a metabolomic analysis to identify potential molecules derived from the CYN was conducted using the Compound discoverer 3.2 software and several databases related to metabolism and natural compounds (Human Metabolome database, BioCyc, FooDB, KEGG, Phenol-explorer and Plant-Cyc, mass bank, Nature chemistry, Food and agricultural organization).

In addition, this same process of CYN extraction in the samples and analysis was performed with untreated rat brain samples (control) spiked with an amount of toxin equivalent to 0.75, 7.5 and 75.0 µg/kg of CYN to check that the extraction and determination procedure could detect CYN in brain samples. In this sense, CYN was detected at all concentrations tested under the conditions employed.

### Statistical analysis

Graph-Pad Prism 8.0.1 Software (Graph-Pad Prism 8 Software Inc., La Jolla, CA, USA) was used for the statistical analysis and graphing. To verify the distribution of the results, Kolmogorov–Smirnov was employed. For data with a normal distribution, the data were analysed using analysis of variance (ANOVA) followed by Dunnett’s multiple comparisons. Exposed animals were compared with the negative control. The results were expressed as mean ± standard deviation (SD). Results were considered statistically significant between *p < 0.05 and ****p < 0.0001.

## Results

### AChE activity

**Fig. 1:**
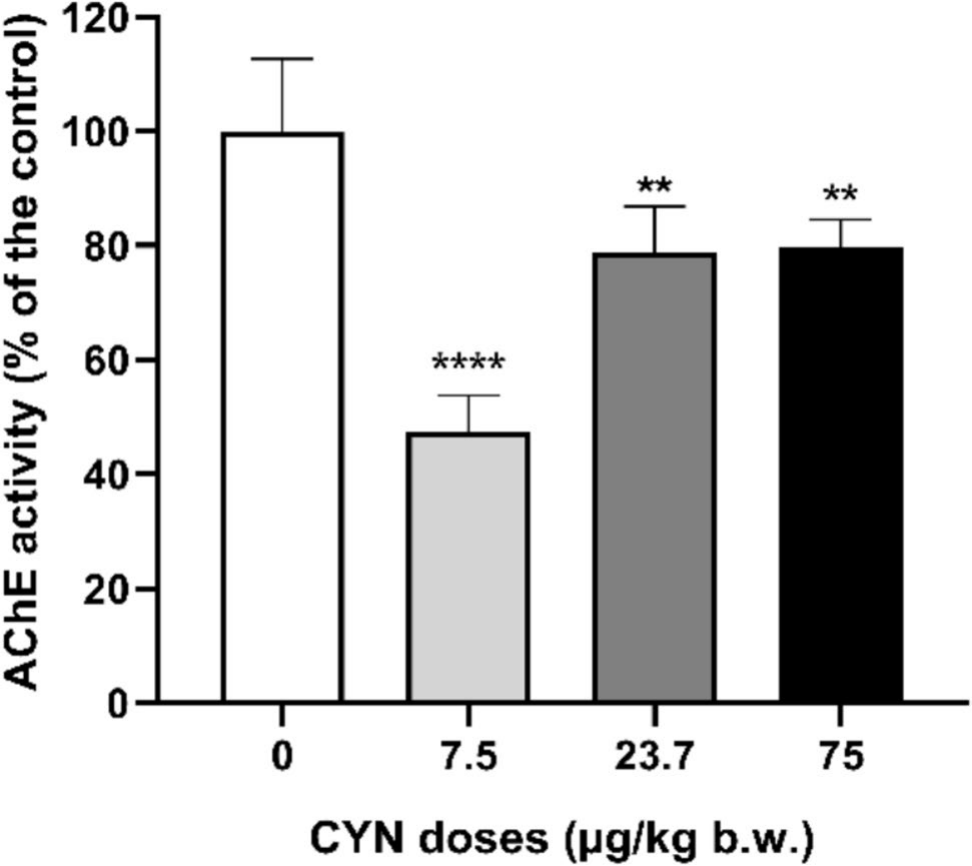
AChE activity in brain of male Wistar rats exposed orally to doses of pure CYN (0–75 µg/kg b.w.). The activity is expressed as % of the control. The significance levels observed are **p < 0.01 and ****p < 0.0001 with respect to the control group

The AChE activity of the brain of male rats exposed to pure CYN is shown in Fig. 1. A significant decrease in the enzymatic activity was observed after exposure to all CYN doses tested compared to the untreated control group. Specifically, in comparison to the control group, enzyme activity was 48% at the lowest dose, whereas 79% and 80% of AChE activity were maintained in rats treated with 23.7 and 75.0 µg/kg b.w., respectively.

### Oxidative stress parameters

**Fig. 2:**
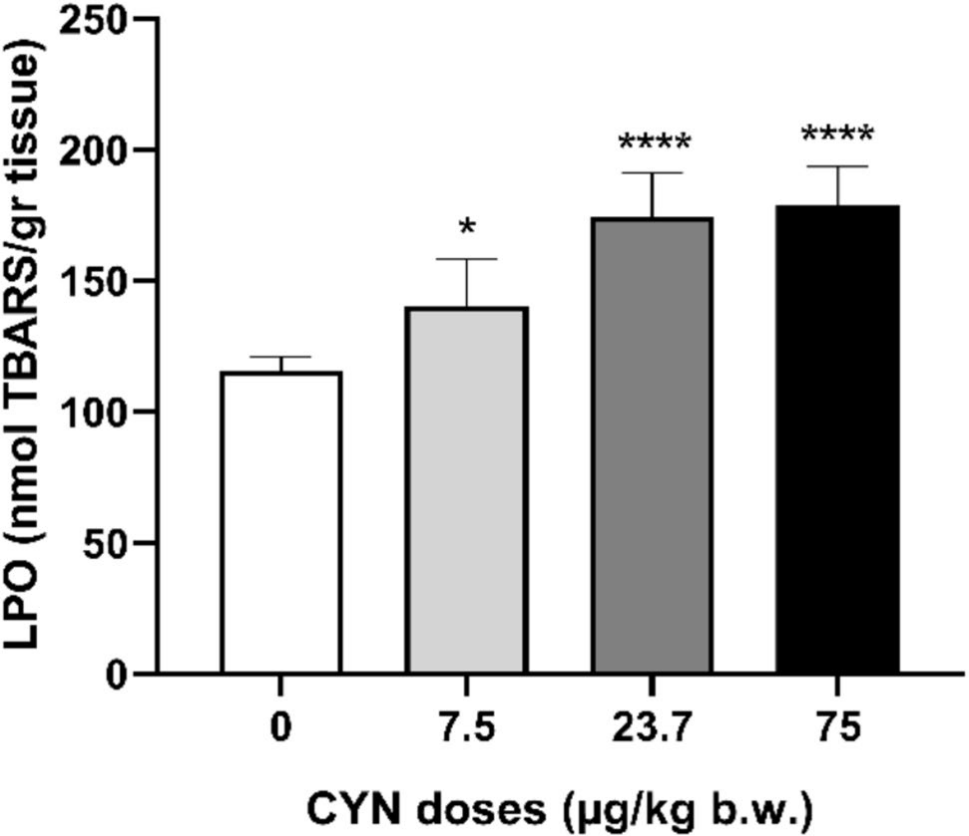
LPO levels in brain of male Wistar rats exposed orally to doses of pure CYN (0–75 µg/kg b.w.). LPO values are expressed as nmol TBARS/gr tissue. The significance levels observed are *p < 0.05 and ****p < 0.0001 in comparison to the control group

Results showed a significant dose-dependent increase in LPO levels (Fig. 2) in the brain of male Wistar rats exposed to CYN by gavage. Specifically, the observed increase was 1.2-fold, 1.5-fold and 1.6-fold for the groups treated with 7.5, 23.7 and 75 µg/kg b.w. versus the control group, respectively.

**Fig. 3:**
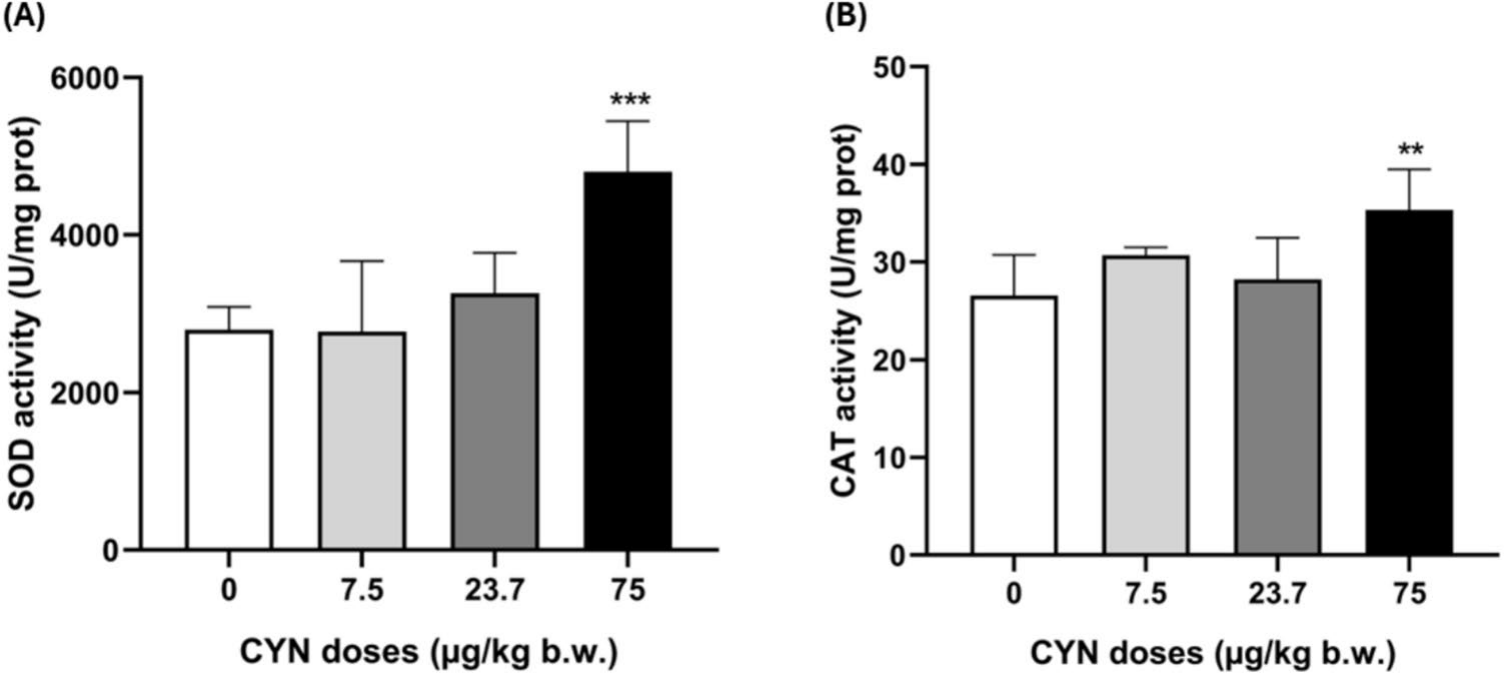
SOD (**A**) and CAT (**B**) activities in brain of male Wistar rats exposed orally to doses of pure CYN (0–75 µg/kg b.w.). Enzymatic activity values are expressed as U/mg protein. The significance levels observed are **p < 0.01 and ***p < 0.001 in comparison to the control group

Regarding SOD and CAT antioxidant enzymatic activities, a significant increase (1.7-fold and 1.3-fold, respectively) was observed in the brain of rats exposed only to the highest dose tested (75 µg CYN/kg b.w.) in both cases (Fig. 3).

**Fig. 4:**
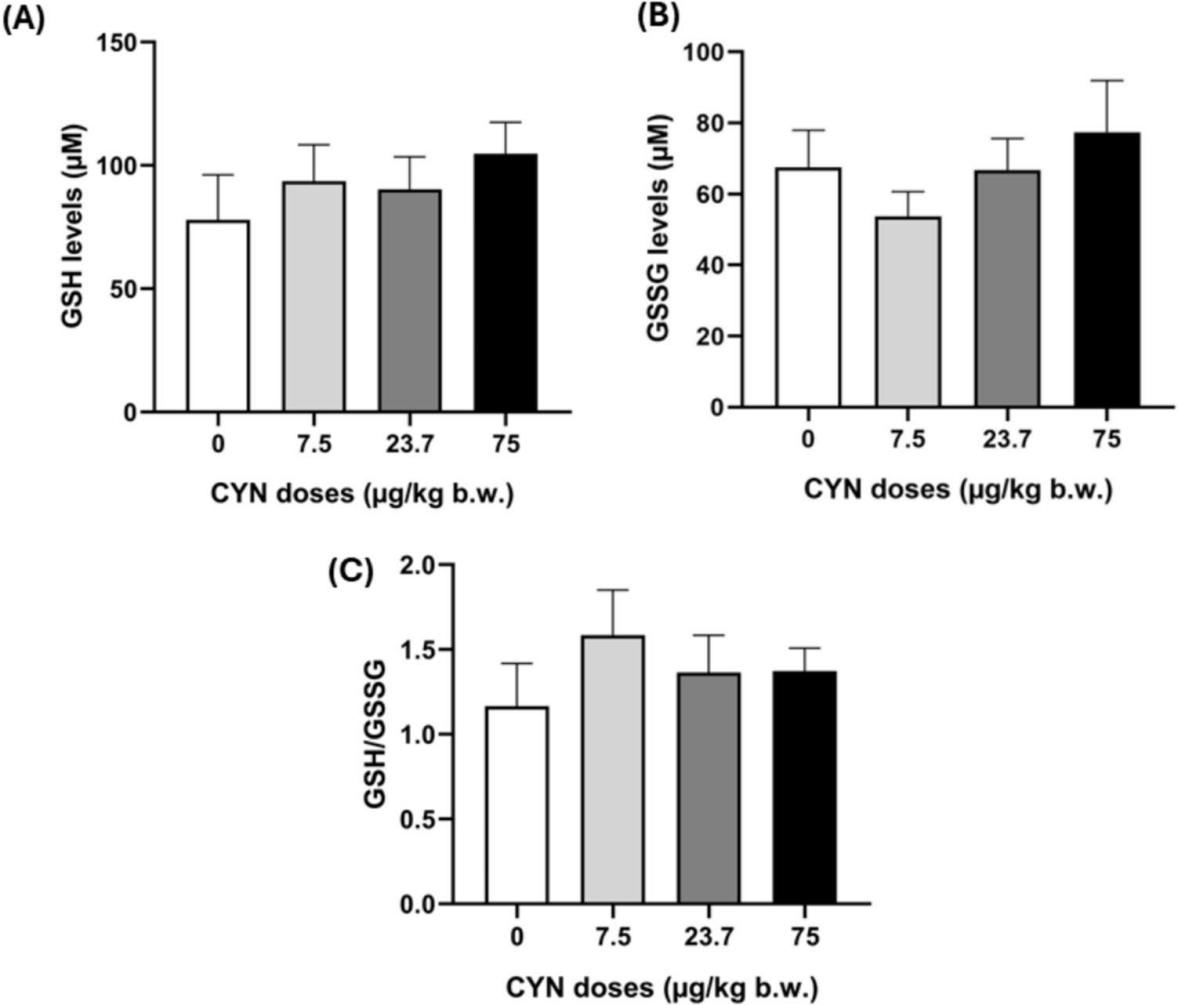
GSH levels (**A**), GSSG levels (**B**) and GSH/GSSG (**C**) ratio in brain of male Wistar rats exposed orally to doses of pure CYN (0–75 µg/kg b.w.)

Regarding GSH and GSSG values, no alterations in the GSH/GSSG ratio were observed at any of the doses assayed (Fig. 4).

### Determination of CYN and its metabolites in rat brain

CYN was not detected in any of the brain samples of rats exposed to the toxin (groups exposed to 7.5, 23.7 and 75.0 µg/kg b.w.). However, a total of 14 potential CYN-derivate compounds were detected and identified in brain extracts of rats exposed to this cyanotoxin (Table 1).

**Table 1:** Potential CYN-derived compounds in brain samples from rats orally exposed to different doses of CYN (7.5, 23.7 and 75.0 µg/kg b.w.)

CYN-derivated compound	Biotransformations	Composition change	m/z	Retention time (min)	7.5 µg/kg b.w		23.7 µg/kg b.w	75 µg/kg b.w	
C_17_H_22_N_6_O_7_S_2_	Dehydration, dehydration, taurine conjugation	+ (C_2_HNS)	509.08815	0.812	Detected	Detected			Detected
C_31_H_51_N_5_O_6_S	Desaturation, nitro reduction, palmitoyl conjugation	− (O) + (C_16_H_30_)	622.36318	7.629	Detected	Detected			Detected
C_18_H_24_N_6_O_7_S_2_	Dehydration, dehydration, cysteine conjugation 2	+ (C_3_H_3_NS)	523.10367	0.820	Detected	Detected			Detected
C_21_H_33_N_9_ O_3_	Nitro reduction, arginine conjugation	− (O_4_ S) + (C_6_H_12_N_4_)	482.25897	9.824	n.d	Detected			Detected
C_15_H_19_N_5_O_8_	Desaturation, thiourea to urea	− (H_2_S) + (O)	398.13034	6.31	Detected	Detected			n.d
C_15_H_19_N_5_O_8_	Dehydration, thiourea to urea	− (H_2_S) + (O)	398.13038	6.162	Detected	Detected			n.d
C_21_H_27_N_5_O_14_	Desaturation, thiourea to urea, Glucuronide conjugation	−(S) + (C_6_H_6_O_7_)	574.16107	3.400	Detected	Detected			n.d
C_15_H_22_N_4_O_10_	Oxidative deamination to alcohol, thiourea to urea	− (NS) + (HO_3_)	419.14029	1.262	n.d	Detected			Detected
	Hydration, oxidative deamination to alcohol, thiourea to urea								
C_20_H_33_N_7_O_9_	Reduction, thiourea to urea, ornitine conjugation	− (S) + (C_5_H_12_N_2_O_2_)	538.22387	5.524	n.d	Detected			Detected
C_16_H_21_N_5_O_4_	Methylation	− (O_3_S) + (C)	348.16636	6.135	Detected	n.d			n.d
C_21_H_25_N_5_O_11_S	Dehydration, dehydration, glucuronide conjugation	+ (C_6_H_4_O_4_)	556.13312	0.811	Detected	n.d			n.d
C_17_H_25_N_5_O_10_S	Hydration, acetylation	+ (C_2_H_4_O_3_)	492.13931	5.522	n.d	n.d			Detected
	Oxidative deamination to alcohol, glycine conjugation								
	Hydration, oxidative deamination to alcohol, glycine conjugation								
C_16_H_21_N_5_O_8_	Desaturation, thiourea to urea, methylation	− (S) + (CO)	412.14587	7.039	Detected	n.d			n.d
C_20_H_26_N_6_O_11_	Oxidative deamination to ketone, thiourea to urea, glutamine conjugation	− (S) + (C_5_H_5_NO_4_)	549.15448	5.101	n.d	n.d			Detected

*n.d.* not detected

Neither CYN nor any of the potential CYN-derived compounds were detected in the brains of the rats in the negative control. A similar number of derivate compounds was detected in all groups exposed to CYN (8–9 compounds/group). Among CYN derivatives detected, only 3 were present in all CYN-exposed groups (C_17_H_22_N_6_O_7_S_2_, C_31_H_51_N_5_O_6_S, C_18_H_24_N_6_O_7_S_2_) (the proposal structure has been shown in supplementary material Table S1). In addition, 3 compounds were only present in the groups exposed to the highest (75.0 µg CYN/kg b.w.) and intermediate (23.7 µg CYN/kg b.w.) doses, 3 compounds exclusively in the groups exposed to the intermediate (23.7 µg CYN/kg b.w.) and low doses (7.5 µg CYN/kg b.w.) and no CYN-derived compound in common was detected only at the highest and lowest dose.

As detailed in Table 1, these potential metabolites of CYN could appear through several alternative routes and as a consequence of the following reactions of Phase I: hydration, oxidative deamination to alcohol, nitro reduction, thiourea to urea, dehydration, desaturation, oxidative deamination to ketone, reduction and reactions of Phase II such as acetylation, methylation or conjugation reactions with glycine, palmitoyl, ornitine, glutamine, cysteine, taurine, arginine and glucuronide.

## Discussion

While the hepatotoxic and cytotoxic properties of CYN have been extensively documented, its neurotoxic potential remains less documented (Yang et al. 2021) even though its chemical structure is more like that of neurotoxins than hepatotoxins (Hinojosa et al. 2019a). Currently, very few studies have explored the neurotoxic effects of this cyanotoxin in both in vitro and in vivo models (Guzmán-Guillén et al. 2015a; Takser et al. 2016; Hinojosa et al. 2019a, 2022, 2019b; Rabelo et al. 2021). Furthermore, most of these studies have been performed with cyanobacterial cultures or extracts in aquatic models. In this sense, increasing evidence suggests that cyanobacterial extracts without known cyanotoxins can exhibit toxicity to the peripheral and central nervous system (CNS) (Metcalf et al. 2021). For this all, it is necessary to investigate the neurotoxic potential of pure CYN to relate the neurotoxic effects directly to this cyanotoxin and not to other compounds.

In this regard, this work addresses, for the first time, the neurotoxic effects of pure CYN in rats orally exposed, specifically focusing on AChE activity and oxidative stress parameters in brain samples. The findings suggest that exposure to pure CYN induces a significant reduction of AChE activity in the brain of exposed rats at all exposure doses (7.5, 23.7 and 75.0 µg/kg b.w.) and no dose-dependent pattern was observed. Consistent with our results, Guzmán-Guillén et al. (2015a) showed a significant decrease in this biomarker in the brain of tilapia fish exposed to a CYN-producing *A. ovalisporum* culture (10 µg/L) by immersion after 14 days. Similarly, in in vitro studies carried out in differentiated SH-SY5Y cells treated with pure CYN (0.075–0.3 µg/ml) for 24 h a significant decrease in AChE activity was also observed (Hinojosa et al. 2019b). On the contrary, in fish (*H. malabaricus*) exposed i.p. to 50 µg/kg b.w. of CYN for 7 and 14 days, Da Silva et al. (2018) only observed a significant increase in the activity of AChE in fish brain after exposure to CYN-producing culture extracts, whereas no significant change after exposure to pure CYN was reported. Although AChE is recognized as a specific biomarker of toxicant exposure (Pretto et al. 2010), contradictory results of this biomarker have been obtained in the brain of animals exposed to CYN in different in vivo assays. This could be due to the use of cyanobacterial extracts or cultures that may contain different compounds with the potential to modify the toxic effect of CYN.

Our study is the first to show a significant decrease in AChE activity produced by pure CYN in the brain of rats. This is important because when this biomarker is inhibited, an accumulation of the transmitter acetylcholine occurs at the nerve synapse, altering the normal function of the nervous system (Capó et al. 2022). These inhibitory effects can lead to physiological alterations, including altered behavior and reduced motility because of their influence on the CNS and neuromuscular junctions (Brandts et al. 2018; Solé et al. 2024). In general, the percentage of inhibition of AChE activity conditions the severity of toxic effects as a consequence of the degree of overstimulation of postsynaptic neurons. The present study shows a decrease in AChE activity of 48% at doses of 7.5 µg/kg bw. Considering the Food and Agriculture Organization (FAO 2007), a reduction in AChE activity of 20% is considered the point at which an anticholinesterasic agent may be harmful; symptoms and signs appear above 50% inhibition, and mortality can occur after 90%. In this study, inhibition of enzyme activity was 48%, 21%, and 20% in rats treated with 7.5, 23.7 and 75.0 µg/kg b.w., respectively. Nevertheless, no signs were detected outside the normal physiological behavior and appearance of the animals that were observed during the whole treatment. In contrast, Saker et al. (2003) observed neurological signs such as piloerection, lethargy or difficulty breathing in mice after i.p. administration of different strains of *C. raciborskii*. However, these authors did not detect CYN in HPLC analyses, so the reported signs may not be caused by this toxin.

In addition, results showed oxidative stress in the brain of rats, as evidenced by altered oxidative stress biomarkers. In this respect, a dose-dependent increase in the brain LPO levels of rats orally exposed to CYN has been observed in this study. These results are consistent with those reported by Guzmán-Guillén et al. (2015a), who observed a significant increase in brain LPO levels after exposing tilapia fish for 14 days to repeated concentrations of CYN-producing *A. ovalisporum* (10 µg/L) by immersion. Similar findings were obtained by Da Silva et al. (2018), in *Hoplias malabaricus* i.p. exposed to a single dose of pure CYN and an extract of *C. raciborskii* (50 µg/kg b.w.). Specifically, these authors noted an increase in LPO levels 7 and 14 days after exposure to pure CYN and CYN extract. Lipids, such as fatty acids, play important functional and structural roles, so the balance and regulation of these molecules is essential for the correct function of the CNS (Solé et al. 2024). Therefore, the increase in LPO produced by CYN in the brain on the one hand could alter the structure of this organ, modify its permeability and promote the flow of other toxins, and, on the other hand, it could alter the normal functions of the brain and lead to significant neurological disorders.

In general, the results show an inverse relationship between LPO levels and AChE enzyme activity in the rat brains exposed to CYN. This same relationship has been described by other authors in fish exposed to CYN and other different xenobiotics such as pesticides or cadmium (Üner et al. 2006; Pretto et al. 2010). The brain requires a high percentage of oxygen from the organism, which makes it more susceptible to oxidation reactions and damage to different molecules, as shown by increased LPO levels. Damage to lipid molecules can interfere with membrane structure and fluidity and affect membrane-bound enzymes such as AChE enzyme activity.

In relation to SOD and CAT enzyme activities, our results show a significant increase only at the highest concentration tested (75 µg CYN/kg b.w.). There are no previous results for these biomarkers in the brains of CYN-exposed animals. These enzymes are the first line of defense against oxidative stress, and their increase indicates that the brain is activating its protection mechanisms against the increase in oxidations produced by CYN exposure in rats. However, under test conditions, SOD and CAT activities were not sufficient to prevent the generation of LPO. Furthermore, despite the need for neutralization of the lipid peroxides produced, no changes in GSH and GSSG levels were found in the brain of rats exposed to CYN for 48 h. In contrast, Da Silva et al. (2018) reported an alteration in GSH levels by CYN exposure in fish brain for 7 and 14 days. According to our results, Hinojosa et al. (2019b) reported no changes in GSH levels in the human neuroblastoma cells line (SH-SY5Y) exposed to 0.25–1 µg/mL of pure CYN after 4, 8, 12 and 24 h. In this regard, despite the main recognized mechanism of action of CYN is the suppression of protein synthesis and GSH, rapid toxicity seems to be driven by metabolites generated by CYP450 (Runnegar et al. 1994; Humpage et al. 2005) and long-term toxicity is because of the inhibition of protein synthesis (Zegura et al. 2011). Therefore, the lack of response in GSH levels in this study may be due in part to the exposure times employed. On the other hand, the first antioxidative pathway consists of SOD followed by CAT activities, which actin parallel to glutathione peroxidase (GPx) as hydroperoxide scavengers. In this case, CAT activity may be sufficient to control these oxyradicals and GSH is not consumed by GPx. Furthermore, no GSH conjugation reactions were observed in the suggested metabolic pathways involved in the production of potential CYN derivatives.

To relate the effects observed in the brain of rats to the exposure to pure CYN, this study analyzed both CYN and its possible metabolites in the brain of Wistar rats exposed to this toxin by UHPLC-MS/MS. The results show that CYN is not detected in the brain of rats exposed to any of the doses. To date, only two studies have detected CYN in the brain of fish exposed to CYN (Guzmán-Guillén et al. 2015a; Da Silva et al. 2018). However, these authors used an ELISA analysis to determine the toxin, and it is possible to observe false positives with these commercial cylindrospermopsin ELISAs (Metcalf et al. 2017). In addition, our results support the argument that CYN is likely to fail to cross the blood–brain barrier through passive diffusion because of its hydrophilic properties (Banks 2009). However, further research is needed to investigate the transport and presence of CYN in the brain (Metcalf et al. 2021).

In the present work, although CYN has not been detected in brain samples, up to 14 compounds have been determined as potential metabolites of this cyanotoxin. This fact could be explained with two assumptions: (1) CYN does not enter the brain and its metabolites formed in other metabolizing organs (such as liver, kidney, etc.) are the ones that pass the blood–brain barrier and/or (2) CYN can enter the brain where it is completely metabolized. In any of these situations, the observed alterations in both oxidative stress parameters and AChE activity can be related to the presence of potential CYN metabolites in the brain of rats orally exposed to pure CYN. In fact, other authors have shown a reduction in CYN toxicity in the presence of metabolism-inhibiting agents in HepaRG cells (Kittler et al. 2016).

In the present work, different metabolic pathways were observed to be involved depending on the exposure doses of CYN under the condition tested. This can be due to the fact that higher CYN doses may lead to saturation of some major metabolic pathways, requiring the activation of a greater number of alternative routes to eliminate the toxin. Thus, a total of 18 reactions have been described in the different metabolic pathways proposed. The most frequent reactions are conjugation or phase II reactions, which account for 30% of all reactions that occur by the various metabolization routes proposed for CYN. Up to 10 different substrates are used for conjugation, most notably conjugation with glycine and glucuronide. To date, reactions of CYN phase II metabolism have not been explored (WHO 2020). Besides, in terms of their occurrence in the proposed metabolic pathways, the thiourea to urea step (19%), dehydration (16%), oxidative deamination (12%), reduction (7%) and hydration (7%) should be highlighted. Similar reactions were detected in CYN-producing *Chrysosporum ovalisporum* extract (Hinojosa et al. 2023) using the same method of determination. However, less complex biotransformation pathways were observed in the cyanobacterial culture extract than in the brain samples. Likewise, Kittler et al. (2016) reported that metabolic activation through phase I metabolism plays only a minor role (approximately 9%) in CYN toxicity but did not investigate phase II reactions. Moreover, the metabolites detected in brain samples of rats exposed to the highest doses of CYN were those derived from the most complex metabolic routes. This is the case of C_15_H_22_N_4_O_10_ and C_17_H_25_N_5_O_10_S derivatives, in which different metabolic pathways could be involved in the same metabolite production. However, this study is limited by the lack of commercially available standards to quantify the detected potential CYN metabolites. This challenge often occurs when working with metabolites and compounds that have not been widely documented in scientific literature, as seen in this particular study. Therefore, the identification of these metabolites can only be done by using libraries of reference spectra. Nevertheless, based on the results obtained in this work, potential metabolites generated by phase I and II reactions could play an important role in CYN toxicity in the nervous system.

## Conclusions

AChE activity decreased in the brain of rats orally exposed to pure CYN at 7.5, 23.7 and 75.0 µg/kg b.w. for 48 h. Moreover, oxidative stress is evidenced by elevated SOD and CAT enzymatic activities and a significant increase in LPO levels, although no changes in GSH levels were detected. These alterations could be directly induced by the presence of potential CYN metabolites from phase I and II biotransformation reactions, described for the first time. Considering that the neurons are highly specialized cells that can be notably vulnerable to the long-term effects of low concentrations of toxic substances, more studies carried out in in vivo mammalian models exposed to pure CYN are essential to be able to investigate the mechanism of CYN toxicity at the nervous system and its potential role in neurodegenerative diseases.

## Supplementary Information

Below is the link to the electronic supplementary material.

Supplementary file1 (DOCX 176 KB)

Supplementary file2 (XLSX 46 KB)

## Data Availability

All data generated or analysed during this study are included in this published article and its supplementary information files.
