# Supplementary material for: Alterations in acetylcholinesterase activity and oxidative stress parameters induced by pure cylindrospermopsin in brain of orally exposed rats and determination of potential metabolites
Source: Arch Toxicol. 2025 Apr 13;99(8):3297–308. doi: 10.1007/s00204-025-04057-5 (PMC12367855; doi:10.1007/s00204-025-04057-5)
Supplement: Supplementary file 1 — Supplementary file1 (DOCX 176 KB) [file 204_2025_4057_MOESM1_ESM.docx]

**Table S1:** Proposed potential main CYN-derived compounds found in brain samples from rats orally exposed to CYN. Proposed molecular structure considering the physico-chemical characteristics described by the Compound discoverer 3.2 software and elaborated with Chemsketch.

| **CYN-derivated compound** | **Biotransformations** | **Composition Change** | **Calculate MW** | **m/z** | **Retention Time (min)** | **Proposed CYN-derived compound** |
| --- | --- | --- | --- | --- | --- | --- |
| C_17_H_22_N_6_O_7_S_2_ | Dehydration, Dehydration, Taurine Conjugation | + (C_2_HNS) | 486.09893 | 509.08815 | 0.812 | 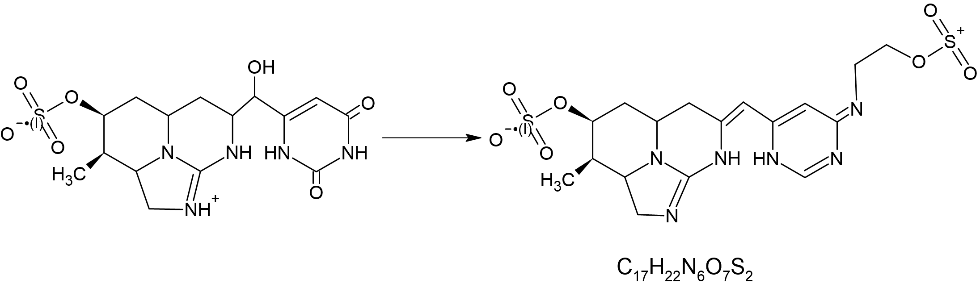 |
| C_31_H_51_N_5_O_6_S | Desaturation, Nitro Reduction, Palmitoyl Conjugation | - (O) + (C_16_H_30_) | 621.3559 | 622.36318 | 7.629 | 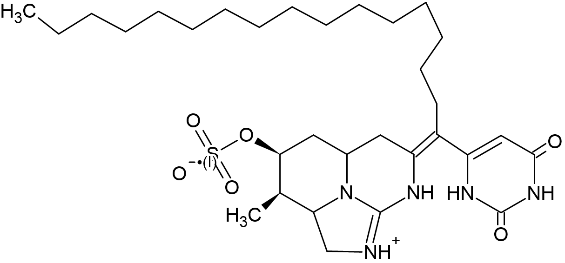 |
| C_18_H_24_N_6_O_7_S_2_ | Dehydration, Dehydration, Cysteine Conjugation 2 | + (C_3_H_3_NS) | 500.11445 | 523.10367 | 0.820 | 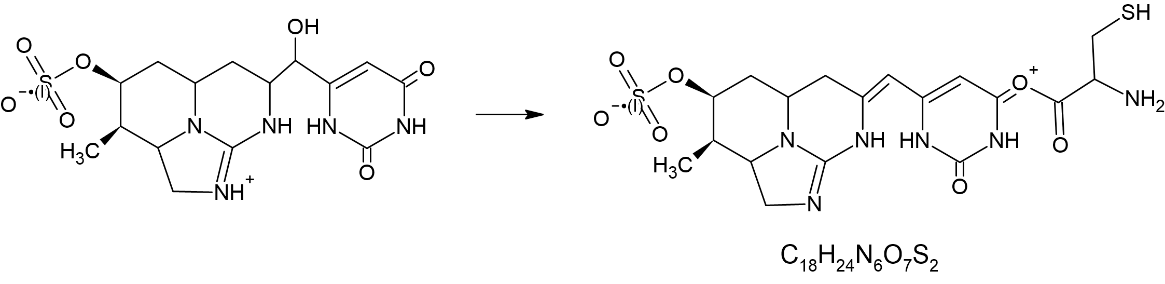 |
